# Effect of tangeretin on cisplatin‐induced oxido‐inflammatory brain damage in rats

**DOI:** 10.1111/jcmm.18565

**Published:** 2024-07-23

**Authors:** Betul Cicek, Betul Danisman, Ismail Bolat, Metin Kiliclioglu, Mehmet Kuzucu, Halis Suleyman, Konstantinos Tsarouhas, Aristidis Tsatsakis, Ali Taghizadehghalehjoughi

**Affiliations:** ^1^ Department of Physiology, Faculty of Medicine Erzincan Binali Yildirim University Erzincan Turkey; ^2^ Department of Biophysics, Faculty of Medicine Ataturk University Erzurum Turkey; ^3^ Department of Pathology, Faculty of Veterinary Atatürk University Erzurum Turkey; ^4^ Department of Biology, Faculty of Arts and Sciences Erzincan Binali Yildirim University Erzincan Turkey; ^5^ Department of Medical Pharmacology, Faculty of Medicine Erzincan Binali Yildirim University Erzincan Turkey; ^6^ Department of Cardiology University General Hospital of Larissa, Terma Mazourlo Larissa Greece; ^7^ Department of Forensic Sciences and Toxicology, Faculty of Medicine University of Crete Heraklion Greece; ^8^ Department of Medical Pharmacology, Faculty of Medicine Bilecik Şeyh Edebali University Bilecik Turkey

**Keywords:** anti‐inflammatory, antioxidant, cisplatin, oxidant/antioxidant status, tangeretin

## Abstract

Cisplatin (CIS) is a platinum‐derived chemotherapeutic agent commonly utilized in the treatment of various malignant tumours. However, anticancer doses of the drug cause serious damage to the brain. This study aimed to determine the potential protective effects of tangeretin, which has antioxidant and anti‐inflammatory properties, in cisplatin‐induced neurotoxicity on BALB/c mice brains. Male BALB/c mice were randomized and separated into four groups. Tangeretin was given for 10 days by gavage. CIS was injected as a single dose of 10 mg/kg intraperitoneally (ip) on the 10th day. Brain tissues, malondialdehyde (MDA), total glutathione (tGSH), glutathione peroxidase (GPx), superoxide dismutase (SOD), catalase (CAT) and nitric oxide (NO) levels were measured to determine oxidative damage and myeloperoxidase, tumour necrosis factor‐alpha (TNF‐α), interleukin 1 beta (IL‐1β), IL‐6 and IL‐10 were measured to determine inflammatory activity. In addition, 8‐OHdG and caspase‐3 were analysed by immunofluorescence methods. While CIS administration remarkably elevated reactive oxygen species, MDA, and NO levels in brain tissue compared to the control, tGSH, GPx, SOD and CAT levels were significantly decreased. Also, it has been detected that TNF‐α, IL‐1β and IL‐6 obtained in CIS‐treated groups increased as well as IL‐10 decreased, thereby elevating the inflammatory response. In addition, 8‐OHdG and caspase‐3 immunoreactivity in neurons increased with CIS administration. Treatment with tangeretin ameliorated the deterioration in oxidant/antioxidant status, overpowered neuroinflammation and ameliorated neurotoxicity‐induced apoptosis. This study shows that tangeretin has beneficial effects on CIS‐induced neurodegeneration. Possible mechanisms underlying these beneficial effects include the antioxidant and anti‐inflammatory properties of tangeretin.

## 1 INTRODUCTION

Cisplatin (CIS) (*cis*‐diamminedichloroplatinum II) is a platinum‐based chemotherapeutic agent on the World Health Organization's list of essential drugs. It is widely used in the treatment of various malignant tumours, including lung, testicular, ovarian and bladder carcinomas.^1^ Although the anticancer effect of CIS cannot be fully explained, it forms crosslinks by binding to DNA in tumour cells and stops DNA synthesis and replication.^2^ However, serious side effects and the phenomenon of drug resistance limit the clinical use of CIS.^1^, ^3^ It has been reported that serious adverse events, including testicular, ototoxicity, nephrotoxicity and cardiotoxicity, have developed in patients treated with CIS.^4^, ^5^ Studies have revealed that neurotoxicity is one of the most far‐reaching problems related to CIS chemotherapy, and neurotoxicity develops in approximately 50% of patients.^1^, ^2^, ^3^ Neurotoxicity may lead to the reduction of the CIS dose and discontinuation of treatment.^6^ However, despite the early discontinuation of treatment, patients still experience neurotoxic symptoms and these symptoms may persist for a long time.^1^, ^3^

The cellular and molecular mechanisms underlying the neurotoxicity of CIS are still not fully understood. Nevertheless, however, it has been shown that CIS causes oxidative damage by increasing malondialdehyde (MDA) levels in nerve cells and decreasing antioxidant levels such as glutathione (GSH), superoxide dismutase (SOD) and catalase (CAT).^7^, ^8^ At the same time, increased reactive oxygen species (ROS) react with DNA and cause oxidative mitochondrial DNA damage.^3^ It has been announced that DNA damage resulting from the activity of CIS exacerbates the neurotoxic process by triggering the activation of proinflammatory cytokines and apoptosis.^9^ Studies on the toxic effects of CIS in the nervous system suggest that agents to increase the repair capacity of cellular DNA damage may supply a promising therapeutic strategy to ameliorate the side effects of CIS.

The protective effects of tangeretin, which is commonly found in citrus peels and a flavonoid in the pentametoxyflavone structure, against oxide‐inflammatory brain damage caused by CIS have been investigated.^10^ Thanks to their antioxidant properties, flavonoids are widely used to alleviate the side effects of chemotherapeutic drugs.^1^, ^3^, ^9^ As a result of ongoing in vivo and in vitro studies, data showing the neuroprotective properties of tangeretin are available.^11^ It has also been reported that tangeretin prevents neuronal apoptosis by suppressing oxidative stress and inflammation.^10^, ^12^ It has been reported that tangeretin protects cerebral ischaemia‐reperfusion by means of its antioxidant and anti‐inflammatory properties.^13^ The knowledge from the literature suggests that tangeretin may protect brain tissue against CIS‐induced oxido‐inflammatory damage. In spite of the antioxidant and anti‐inflammatory properties of tangeretin, no information has been found in the literature regarding its protective effect against brain damage caused by CIS. So, our study aims to biochemically and pathologically explore the effect of tangeretin on CIS‐induced oxide‐inflammatory brain injury in rats.

## 2 MATERIALS AND METHODS

### 2.1 Drugs and chemicals

CIS was acquired from Koçak Farma (50 mg/100 mL vial) (İstanbul, Turkey), and tangeretin was procured from Sigma‐Aldrich (St. Louis, MO, USA). Sevorane liquid 100% was obtained from Abbott Laboratory (Istanbul, Turkey). All other chemicals used during the experiment were of analytical grade and were purchased from Sigma‐Aldrich (St. Louis, MO, USA).

### 2.2 Animals

In the experiment, 24 male BALB/c mice weighing 18–20 g were procured from Atatürk University Medical Experimental Application and Research Center (Erzurum, Turkey). Animals were fed with commercial rat chow and tap water ad libitum in 12 h light/12 h dark cycle at normal room temperature (22 ± 0.5°C). After a 1‐week adaptation period, the experiment was started. The protocols and policies were advocated by the local Animal Experimentation Ethics Committee (date: 25 May 2023, meeting no. E‐36643897‐000‐2300162197).

### 2.3 Experimental design

BALB/c mice were randomly split into four groups, each of the six mice, as a sham (SH), 10 mg/kg CIS alone, 10 mg/kg tangeretin + CIS (TCIS 10), 20 mg/kg tangeretin + CIS (TCIS 20).

#### 2.3.1 Experimental procedure

Tangeretin was administered orally via gastric gavage at doses of 10 mg/kg and 20 mg/kg to TCIS 10 (*n* = 6) and TCIS 20 (*n* = 6) groups of animals, respectively. Tangeretin was dissolved in 0.9% NaCl containing 0.1% dimethylsulfoxide (DMSO) and administered orally. The same volume of 0.9% NaCl enclosing 0.1% DMSO was given orally to the SH (*n* = 6) and CIS (*n* = 6) groups. Tangeretin was given to the animals for 10 days. On the 10th Day, a single ip dose of 10 mg/kg CIS was provided. On Day 11 of the experiment, animals were sacrificed by decapitation under sevoflurane anaesthesia. Afterwards, the brain tissues of the sacrificed mice were removed immediately and split into two sagittal sections washed with cold 0.9% NaCl. One‐half of it was stored in a 10% formalin solution for immunofluorescence analysis. The other half was homogenized for biochemical analysis. Biochemical and immunofluorescence findings obtained from all groups were compared between groups and evaluated.

### 2.4 Biochemical analyses

#### 2.4.1 Preparation of samples

The half part of the brain was weighed and homogenized in cold 0.1 M phosphate buffer (pH = 7.4) solution, 10% tissue homogenate was prepared, and supernatants were obtained by centrifuging at 10,000 rpm at 4°C for 20 min.^14^ MDA, total glutathione (tGSH) and SOD as oxidative stress parameters in supernatant samples; TNF‐α and IL‐1β levels were analysed as markers of inflammation.

#### 2.4.2 Determination of MDA, tGSH and SOD

Determination of MDA concentration is related to the method employed by Ohkawa et al., which is associated with the spectrophotometric determination of the absorbance of the pink‐coloured complex established by thiobarbituric acid (TBA) and MDA at a wavelength of 532 nm.^15^ tGSH measurement was made in compliance with the method described by Sedlak and Lindsay. DTNB [5,5'‐Dithiobis (2‐nitrobenzoic acid)], which is used for the determination of tGSH, is a disulphide chromogen and is simply reduced by compounds with sulfhydryl groups. The yellow colour formed as a result of this reaction is measured spectrophotometrically at 412 nm.^16^ The method used by Sun et al. was used for the determination of SOD activity.^17^ This reaction starts with the oxygen radical (O_2_^−^) reduction of nitroblue tetrazolium (NBT) in the environment. It depends on the determination of the absorbance of formazan, a purple‐coloured compound formed as a result of this reaction, at a wavelength of 560 nm. In the determination of homogenate protein amount, the Lowry et al., method was used.^18^ CAT activity was determined with Aebi's method which is based on the measurement of absorbance reduction due to H_2_O_2_ consumption at 240 nm.^19^ The activity of glutathione peroxidase (GPx) was measured in agreement with Lawrence and Burk's method.^20^ Nitric oxide (NO) concentration was evaluated based on the reaction of nitrite content with Griess reagent to establish a coloured compound that absorbs light at 540 nm.^21^ The protein level in brain homogenate was evaluated chemically with bovine serum albumin as a standard.^22^ ROS concentration was determined with an ELISA kit (LSBio, USA) as specified by the manufacturer's direction.

#### 2.4.3 Determination of inflammatory marker levels

TNF‐α (Cat. No. E‐EL‐M3063), IL‐1β (Cat. No. E‐EL‐M0037), IL‐6 (Cat. No. E‐EL‐M0044) and IL‐10 (Cat. No. E‐EL‐M0046) levels in brain tissues were measured using commercial ELISA kits (Elabscience, USA) according to the manufacturer procedures. Optical density (OD) was quantified spectrophotometrically at 450± 2 nm. The OD value is proportional to the mouse TNF‐α, IL‐1β, IL‐6 and IL‐10 concentrations. Inflammatory cytokines levels in the samples were calculated by comparing the optical density of the samples with the standard curve.

#### 2.4.4 Double immunofluorescence assay

Deparaffinized and dehydrated tissue sections on adhesive (poly‐L‐lysin) slides were used for immunoperoxidase examination. H_2_O_2_ (3%) was then used to inactivate endogenous peroxidase for 10 min. Then the antigen retrieval (citrate buffer (pH + 6.1) 100×) solution was employed to boil tissues and ensured to cool at room temperature. Sections were mounted with protein block for 5 min to arrest nonspecific surrounding staining in tissues. Primary antibody (8‐OHdG Cat. No. sc‐sc‐66036, dilution ratio: 1/100, USA) was dripped onto the tissues and performed in harmony with the instructions for employment. The secondary antibody of immunofluorescence was employed as a secondary marker (FITC Cat. No. ab6785 diluent ratio: 1/1000) and retained in the dark for 45 min. The sections were applied second primary antibody (Caspase 3 Cat. No. sc‐56053, dilution ratio: 1/100, USA) and maintained in conformity with the manufacturer's guidelines. A secondary antibody of immunofluorescence was applied as a secondary marker (Texas Red Cat. No. ab6719 diluent ratio: 1/1000 USA) and held on in the dark for 45 min. DAPI with mounting medium (Cat. no. D1306 dilution ratio: 1/200 USA) was put in the sections, retained in the dark for 5 min, and closed with a coverslip. The stained sections were assessed with a fluorescent microscope (Zeiss Axio, Germany).

### 2.5 Statistical analysis

SPSS 20.00 (IBM SPSS Data Collection, Windows, Inc., Chicago, IL, USA) programme was used for statistical analysis and the results were shown as mean ± standard deviation (SD). The significance was accepted as *p* < 0.05. Parametric ‘one‐way analysis of variance (anova)’ was used for the data conforming to the normality distribution and homogeneity of the variances, and the ‘Tukey post hoc’ test was used for pairwise comparisons. For the data that did not comply with the normality distribution or did not provide the homogeneity of variance, the difference between the groups was determined by the Kruskal–Wallis and the Mann–Whitney *U* test.

## 3 RESULTS

### 3.1 ROS and MDA levels

**FIGURE 1:**
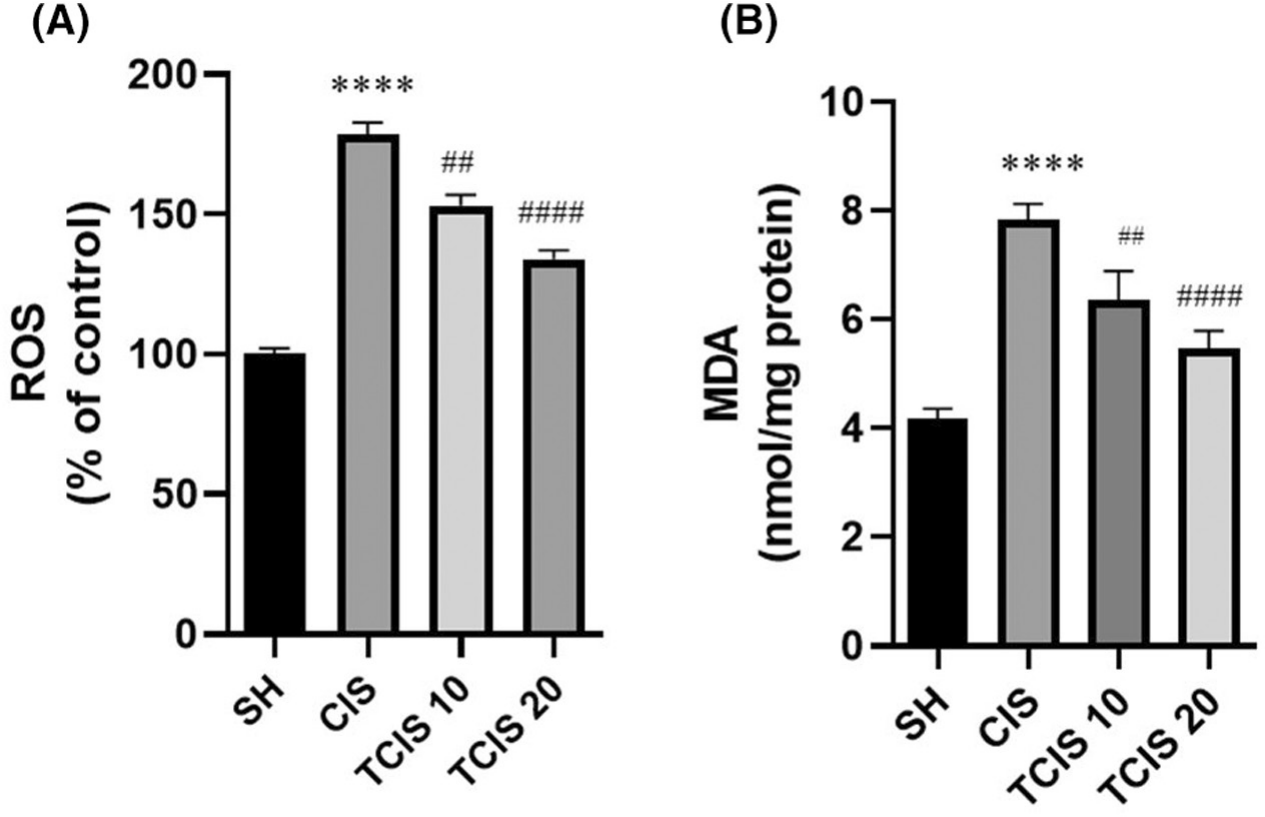
Effects of tangeretin administration on brain's reactive oxygen species (ROS) (A) and MDA (B) level. Data are exhibited as mean ± SD (*n* = 6). ^****^ *p* < 0.0001; significant difference by SH group, ^##^ *p* < 0.01, ^####^ *p* < 0.0001; significant difference by CIS group. SH, sham; CIS, cisplatin; TCIS 10, 10 mg/kg tangeretin + cisplatin; TCIS 20, 20 mg/kg tangeretin + cisplatin.

It was observed that ROS concentration was raised crucially in brain tissue compared to the SH group in the CIS‐treated group (*p* < 0.0001). Treatment of tangeretin decreased the level of ROS compared to the CIS group however, this effect of tangeretin was more effective at 20 mg/kg dose (*p* < 0.0001; Figure 1A).

As can be seen from Figure 1B, CIS administration caused an elevation in MDA in brain tissue (*p* < 0.0001). However, tangeretin (10 mg/kg dose) outstandingly inhibited the rise in MDA with CIS (*p* < 0.01). Tangeretin at 20 mg/kg dose suppressed the increase in MDA with CIS more remarkably than at a dose of 10 mg/kg.

### 3.2 tGSH

**FIGURE 2:**
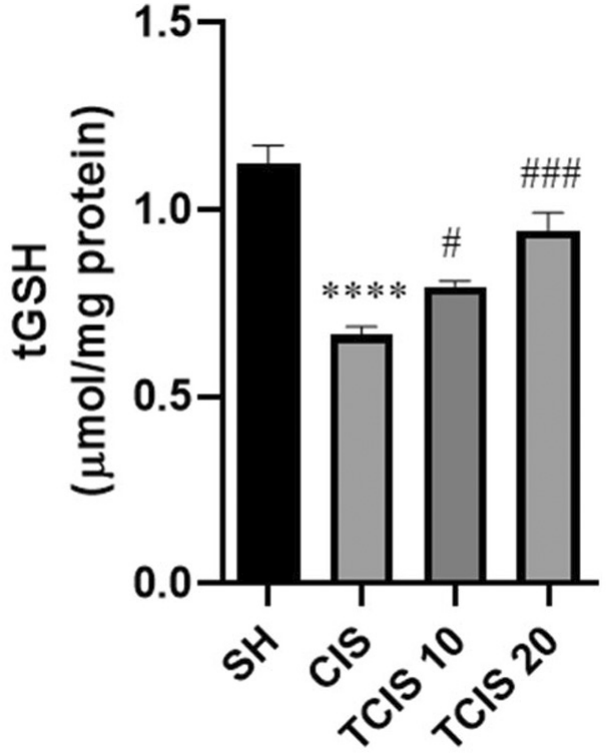
Effects of tangeretin administration on tGSH levels. Data are exhibited as mean ± SD (*n* = 6). ^****^ *p* < 0.0001; significant difference by SH group, ^#^ *p* < 0.05, ^##^ *p* < 0.01, ^###^ *p* < 0.001; significant difference by CIS group. SH, sham; CIS, cisplatin; TCIS 10, 10 mg/kg tangeretin + cisplatin; TCIS 20, 20 mg/kg tangeretin + cisplatin.

tGSH levels in the brain of the CIS‐treated mice were found to be remarkably lower compared to the SH group (*p* < 0.0001) (Figure 2). Tangeretin notably prevented CIS‐induced reduction of tGSH level at 10 mg/kg dose (*p* < 0.05), whereas its effect is more pronounced at a dose of 20 mg/kg (*p* < 0.001).

### 3.3 SOD, CAT and GPx

**FIGURE 3:**
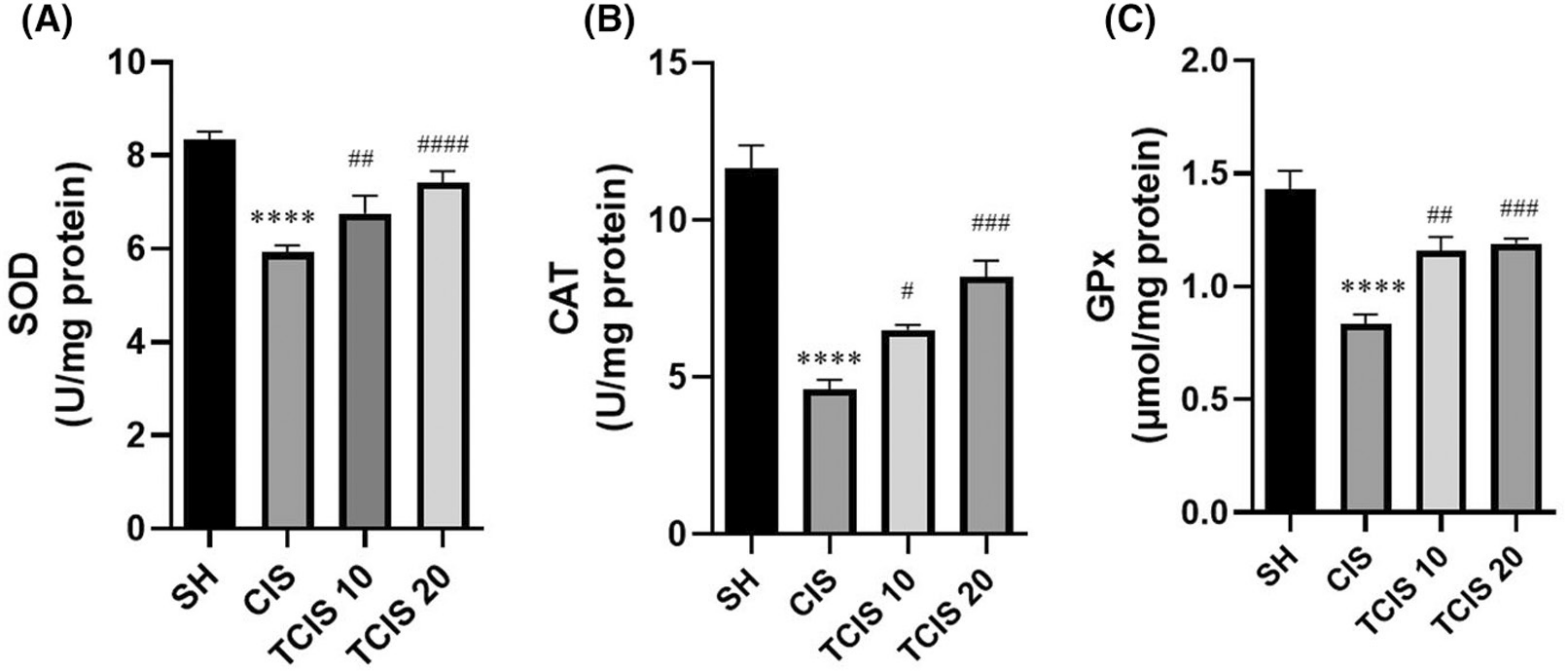
Effects of tangeretin administration on SOD (A), CAT (B) and GPx (C) activities. Data are exhibited as mean ± SD (*n* = 6). ^****^ *p* < 0.0001; significant difference by SH group, ^#^ *p* < 0.05, ^##^ *p* < 0.01, ^###^ *p* < 0.001, ^####^ *p* < 0.0001; significant difference by CIS group. SH, sham; CIS, cisplatin; TCIS 10, 10 mg/kg tangeretin + cisplatin; TCIS 20, 20 mg/kg tangeretin + cisplatin.

The effect of tangeretin treatment on brain SOD, CAT and GPx antioxidant enzyme activities in CIS‐treated mice was demonstrated in Figure 3A–C. SOD, CAT and GPx enzyme activity in the brain of the CIS group was notably diminished compared to the SH group (*p* < 0.0001). It was found that these enzyme activities were markedly higher in the 10 and 20 mg/kg tangeretin treatment groups compared to the CIS group. The group that administered tangeretin at a dose of 20 mg/kg had the highest SOD, CAT and GPx activities in the brain.

### 3.4 NO

**FIGURE 4:**
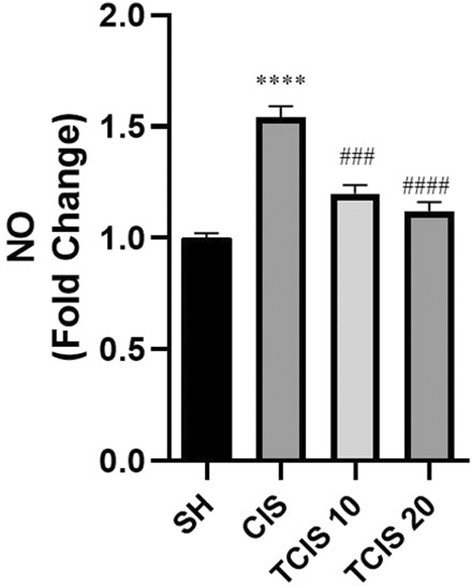
Effects of tangeretin administration on NO levels. Data are exhibited as mean ± SD (*n* = 6). ^****^ *p* < 0.0001; significant difference by SH group, ^###^ *p* < 0.001, ^####^ *p* < 0.0001; significant difference by CIS group. SH, sham; CIS, cisplatin; TCIS 10, 10 mg/kg tangeretin + CIS; TCIS 20, 20 mg/kg tangeretin + CIS.

Administration of CIS provoked a statistically marked elevation in the brain level of NO as compared to the SH group (*p* < 0.0001). This CIS‐induced alteration in the NO status was importantly reversed upon treatment with tangeretin. Treatment with 20 mg/kg of tangeretin (*p* < 0.0001) decreased the concentration of NO most effectively than 10 mg/kg of tangeretin (*p* < 0.001) treatment, compared with the CP group (Figure 4).

### 3.5 Inflammatory markers

**FIGURE 5:**
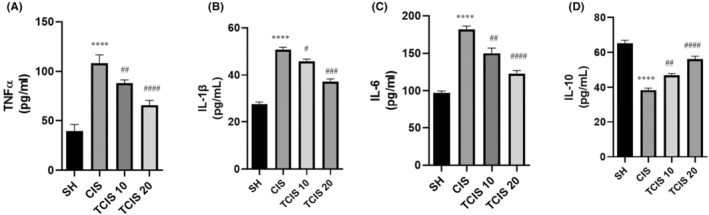
Effects of tangeretin administration on TNF‐α (A), IL‐1β (B), IL‐16 (C) and IL‐10 (D) levels. Data are exhibited as mean ± SD (*n* = 6). ^****^ *p* < 0.0001; significant difference by SH group, ^#^ *p* < 0.05, ^##^ *p* < 0.01, ^###^ *p* < 0.001, ^####^ *p* < 0.0001; significant difference by CIS group. SH, sham; CIS, cisplatin; TCIS 10, 10 mg/kg tangeretin + CIS; TCIS 20, 20 mg/kg tangeretin + CIS.

The concentration of TNF‐α in the brain of the mice in the CIS group was outstandingly higher than in the SH group (*p* < 0.0001) (Figure 5A). Tangeretin seriously inhibited the increment in TNF‐α concentration at a dose of 10 mg/kg with CIS administration (*p* < 0.01). Tangeretin at a dose of 20 mg/kg inhibited the increase in TNF‐α concentration more significantly (*p* < 0.0001).

At the same time, CIS administration caused an increment in IL‐1β and IL‐6 levels in brain tissues (*p* < 0.0001). However, it was found that tangeretin markedly inhibited the increase in IL‐1β and IL‐6 levels with CIS at a dose of 10 mg/kg (*p* < 0.05 and *p* < 0.01, respectively). Tangeretin at a dose of 20 mg/kg suppressed increment in IL‐1β and IL‐6 levels more crucially than CIS at a dose of 10 mg/kg (*p* < 0.001 and *p* < 0.0001, respectively) (Figure 5B,C).

IL‐10 anti‐inflammatory marker was reduced notably in brain tissues in the CIS group compared to the SH group (*p* < 0.0001). However, it was determined that both tangeretin doses increased this anti‐inflammatory marker level compared to the CIS group. Whereas its effect is more pronounced at a dose of 20 mg/kg (*p* < 0.0001) (Figure 5D).

### 3.6 Immunofluorescence

**FIGURE 6:**
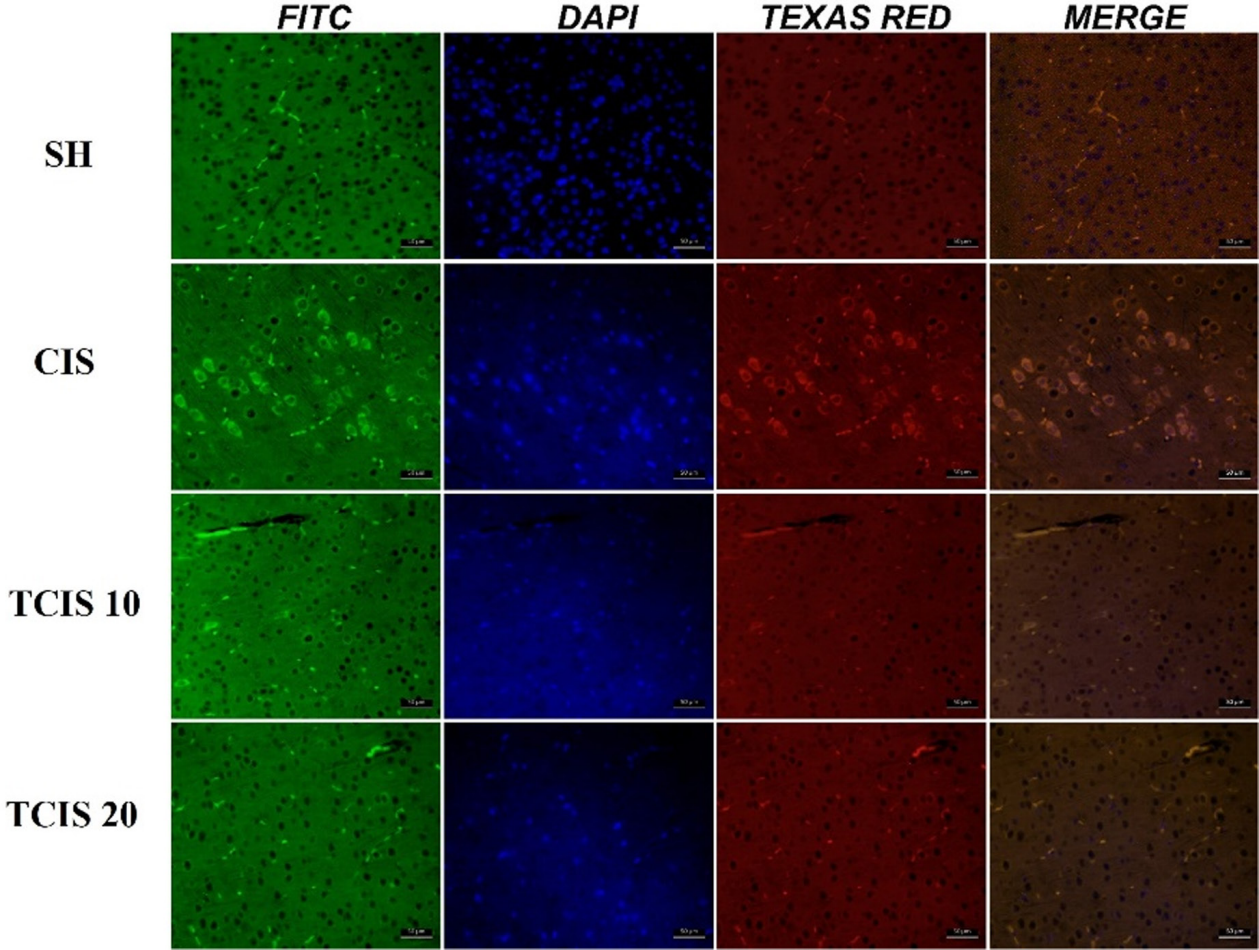
8‐OHdG (FITC) and caspase 3 (Texas Red) expression were observed in neurons in the brain (*n* = 6). The magnification ratio is 20×, scale bar is 50 μm.

8‐OHdG and caspase‐3 immunoreactivities in brain tissues were determined by the immunofluorescence method and a remarkable difference was obtained between the groups (Figure 6 and Table 1). As a result of CIS application, intracytoplasmic 8‐OHdG, and caspase‐3 immunoreactivity in neurons were notably increased compared to the SH group (*p* < 0.0001). Administration of 10 mg/kg tangeretin produced an outstanding reduction in the numbers of 8‐OHdG and caspase‐3 immunoreactive cells (*p* < 0.05). Tangeretin at a dose of 20 mg/kg more significantly inhibited the increase in intracytoplasmic 8‐OHdG and caspase‐3 immunoreactivity in neurons (*p* < 0.001).

**TABLE 1:** 8‐OHdG (FITC) and caspase 3 (Texas Red) expression.

	8‐OHdG	Caspase 3
SH	18.57 ± 0.18	16.42 ± 0.2
CIS	84.49 ± 2.57*	81.13 ± 2.64*
TCIS 10	62.58 ± 1.13**	55.84 ± 1.03**
TCIS 20	31.16 ± 0.49***	26.1 ± 0.28***

*Note*: Data are expressed as the means (SD).

Abbreviations: CIS, cisplatin; SH, sham; TCIS 10, 10 mg/kg tangeretin + cis; TCIS 20, 20 mg/kg tangeretin + CIS.

* *p* < 0.0001 versus the SH group.

** *p* < 0.05 versus the CIS group.

*** *p* < 0.001 versus the CIS group.

## 4 DISCUSSION

Patients treated with CIS many times experience neurotoxic symptoms, which may result in premature discontinuation of treatment.^23^ Combining chemotherapeutic drugs with another protective natural compound is one of the strategies used to alleviate the severity of anticancer drug‐related toxicity.^24^ Several reports conclude that antioxidant compounds including melatonin, erythropoietin, alpha lipoic acid and vitamin E prevent or ameliorate CIS‐related neurotoxicity.^25^, ^26^, ^27^, ^28^ In the current study, the effect of tangeretin on CIS‐induced oxidative brain damage in BALB/c mice was investigated by biochemical and immunofluorescence methods.

Oxidative stress, which results from an excessive formation of ROS and suppression of the antioxidant system, is one of the most significant causes of CIS‐related brain damage.^1^, ^2^, ^7^ It is known that ROS can attack polyunsaturated fatty acids of cellular membranes, resulting in functional and/or structural damage to the membranes and eventually initiate lipid peroxidation (LPO).^29^, ^30^ MDA is the end product of LPO, resulting from lipid peroxide decomposition.^29^ MDA causes cross‐linking and polymerisation of the membrane components, thus exacerbating the damage caused by LPO.^30^ Therefore, increased MDA, a product of LPO, may indicate oxidative stress‐related brain damage.^31^ According to the literature review, the increase in MDA levels in brain tissue is closely related to neuronal degeneration in CIS toxicity.^1^, ^3^, ^7^ Our experimental results showed that CIS significantly increased the ROS and MDA levels in the brain tissue of mice following the literature.^7^, ^32^

Antioxidants are among the most preferred products to reduce or prevent CIS cytotoxicity recently. Therefore, in our study, we investigated the effect of tangeretin, which is known to have antioxidant properties against possible brain damage of CIS. It is reported that tangeretin shows strong antioxidant properties by neutralising free radicals and other ROS, thanks to the four methyl groups in its structure.^10^, ^12^, ^33^ Our study findings showed that tangeretin treatment of mice significantly decreased the CIS‐induced augmentation in ROS and MDA. The role of tangeretin treatment has been previously studied in various experimental brain injuries and supported our findings.^10^, ^12^ Sedik et al., reported that tangeretin showed a protective effect by inhibiting LPO in chromium‐induced acute brain injury in rats.^10^ At the same time, in another study, it was reported that the increased amount of MDA in human brain microvascular endothelial cells due to oxygen‐glucose deprivation was significantly inhibited by tangeretin administration.^12^ Our experimental results and the information obtained from the literature indicate that tangeretin suppresses the excessive ROS production of CIS by preventing the CIS‐induced increase in MDA in brain tissue.

Intracellular redox balance is controlled by many mechanisms, and disruption of this balance has been associated with changes in gene and protein expressions of various antioxidant enzymes.^34^ Measurement of antioxidant enzyme activities, which play a pivotal role in the removal of free oxygen radicals, is one of the methods frequently used to determine brain damage caused by impaired redox balance.^3^, ^9^, ^31^ Therefore, in our study, tGSH, GPx, SOD and CAT antioxidant levels were measured. GSH is the major cellular non‐enzymatic antioxidant and redox regulator in the brain. GSH protects the redox status of the cell by reacting with H_2_O_2_ and organic peroxides and thus shows antioxidant effects.^35^, ^36^ With this, GPx inactivates lipid hydroperoxides as well as H_2_O_2_ via the oxidation of reduced GSH into its disulphide form.^37^ There are many studies documenting a decrease in the amount of GSH and activity of GPx in CIS‐induced oxidative brain damage.^1^, ^3^, ^9^, ^30^ It is thought that the negative effects of LPO and the decrease in the concentrations of GSH and GPx in the brain play an important role in the toxicity of CIS.^1^, ^9^, ^30^ In our study, we found a decrease in the amount of endogenous tGSH with increasing MDA level in the brain of CIS administered mice. In the literature, no studies investigating the effect of tangeretin on decreased tGSH levels and GPx activity in CIS‐induced brain damage were found. However, Fatima et al., argued that tangeretin treatment prevented the decrease in the amount of GSH in brain tissue in the experimental Parkinson's disease.^38^ Another experimental study demonstrated that the administration of tangeretin markedly improves GSH concentration as well as reduces the MDA in hepatic tissue when compared to only the treated CIS group.^39^

NO, a gaseous powerful free radical, reacts with the obtainable superoxide radical to establish peroxynitrite, a more strong oxidant.^40^ This radical can firmly react with DNA, proteins and lipids, as well as suppress the activity of GSH and GPx, and eventually contribute to brain damage.^41^ The participation of NO in the damage of CIS has been declared and ascribed to the enhancement of iNO synthase.^7^, ^41^ In our results, which showed a significant increase in NO levels in the brain of administered CIS, a similar pattern of results was obtained as reported by Aydin et al.^42^ CIS‐induced alteration in NO concentration was remarkably alleviated with tangeretin treatment. The beneficial effect of tangeretin may be mainly attributed to its ability to eradicate free radicals.^12^, ^38^ Evidence supports the formidable radical scavenging activity of tangeretin as being an aromatic ring, a heterocyclic pyran ring, and a methoxy group that can directly react with free radicals.^43^ These satisfying effects of tangeretin may explain its capability to restore the normal oxidant status of the brain in the current report.

In our study, SOD and CAT were the other antioxidants that decreased in the brain tissue of mice as a result of CIS administration. SOD and CAT, which are included in enzymatic antioxidant defence mechanisms, catalyse the conversion of superoxide radicals into H_2_O_2_, resulting in suppression of hydrogen radical production and eventually inhibiting LPO.^10^, ^34^, ^36^, ^44^ Studies have confirmed that an increase in MDA level followed by a decrease in GSH and GPx antioxidant enzyme level along with SOD and CAT is involved in CIS‐associated brain damage.^1^, ^3^, ^7^, ^9^, ^30^ In the present study, the significant decrease in SOD and CAT activities in the brain tissue of CIS‐exposed mice supports this hypothesis, because increased LPO and metabolic failure in antioxidant mechanisms are known to be an important factor for neuronal cell death. However, tangeretin significantly inhibited the CIS‐induced decrease in SOD and CAT activities. Our findings suggest that tangeretin protects brain tissue from CIS‐induced oxidative damage and shows antioxidant activity. In another study, supporting our thoughts about the antioxidant property of tangeretin, it was reported that tangeretin suppressed oxidative stress by preventing LPO and increasing antioxidant capacity, and thus showed neuroprotective effects.^12^

In the literature, it has been shown that ROS causes overproduction of proinflammatory cytokines in nerve cells due to CIS‐induced neurotoxicity.^7^ Among the proinflammatory cytokines, TNF‐α, IL‐1β and IL‐6 are immensely potent and regarded to be the crucial cytokines seeing that they can also initiate a cascade of events and influence the release of other cytokines.^45^ The majority of prior research has demonstrated in the course of central nervous system pathology, IL‐10 concentration rises in the brain to provide nervous tissue survival and ameliorate inflammatory responses activating several signalling pleiotropic pathways.^46^ Data from the literature showed that CIS exposure triggers the release of proinflammatory cytokines TNF‐α, IL‐1β and IL‐6 as well as the reduction of IL‐10 in brain tissue.^7^, ^47^ Therefore, in our study, we investigated the effect of tangeretin on the levels of TNF‐α, IL‐1β and IL‐6 proinflammatory cytokines as well as IL‐10 anti‐inflammatory cytokine in the brain of CIS‐treated mice. The present study confirmed the findings that an increase in TNF‐α, IL‐1β and IL‐6 levels, and a reduction of IL‐10 concentration in brain tissues with CIS administration compared to the control group was associated with the triggering of the inflammatory cascade. However, we found that the tangeretin administration markedly ameliorated the elevation of these proinflammatory cytokine cytokines and suppressed the reduction of IL‐10 anti‐inflammatory cytokines in brain homogenate. In the literature, no study on the effect of tangeretin on CIS‐induced inflammatory processes in brains was found. However, as observed in this study, tangeretin‐mediated inhibition of the increase in inflammatory cytokines levels in brain tissues may be related to the possible anti‐inflammatory and antioxidant activity of tangeretin reported in other studies.^13^, ^48^

As is known, 8‐OHdG is one of the most important biomarkers of oxidative stress and is an important indicator used in the determination of DNA damage.^49^ Khalil et al.,^50^ reported that 8‐OHdG levels were increased and the degree of DNA damage was increased in the CIS group. Similarly, Mansour et al., reported that 8‐OHdG levels increased in the brain tissues of CIS‐treated rats and this was associated with the increase in ROS production caused by CIS and acceleration of oxidative DNA damage.^51^ The immunofluorescence results from this study showed that increased 8‐OHdG immunoreactivity was a good indicator of CIS‐induced neuronal DNA damage in mice. We think that the most probable reason for this situation is the inadequacy of repair defence mechanisms in parallel with the decrease in antioxidant activity. However, in our study, tangeretin showed a protective effect by significantly reducing the level of CIS‐induced oxidative DNA damage by showing antioxidant effects. In previous studies, it was found that DNA damage was reduced with tangeretin supplementation.^12^, ^52^ The results obtained in our study supported these data.

Studies have shown that CIS induces neuronal cell death through mechanisms related to the induction of oxidative stress by proinflammatory cytokines and stimulation of apoptotic factors.^8^ Caspase activation, an important component of apoptotic cell death, has been associated with the destruction of neurons in the brain.^53^ Data from the literature have shown that CIS administration causes caspase‐3 activation leading to apoptosis in the brain and is considered an important parameter in detecting brain damage.^54^, ^55^ In this study, CIS administration to mice significantly increased caspase‐3 immunoreactivity in brain tissue, whereas tangeretin administration caused a significant decrease in caspase‐3 immunoreactivity. The results of this study suggest a possible relationship between inhibition of caspase‐3‐mediated apoptotic signalling and the neuroprotective effect of tangeretin. Studies showing the effect of tangeretin on brain tissue are very limited in the literature. However, our findings are in agreement with Sedik et al., who demonstrated that chromium‐induced apoptotic brain damage associated with caspase‐3 increase was prevented by tangerine.^10^

In conclusion; the effects of tangeretin on brain damage caused by CIS were also evaluated for the first time in this study. Only a limited study of its neuroprotective potential has been published in the literature, and it has been found that tangeretin significantly suppresses oxidative stress, inflammation and activation of apoptotic pathways, which are responsible for the neurotoxic mechanism caused by CIS. The possibility that tangeretin, which has been shown to have antioxidant and anti‐inflammatory properties in previous studies, may reduce side effects such as CIS‐induced brain toxicity suggests that it may be very helpful both in improving the quality of life of patients receiving CIS treatment and ensuring that CIS is used at the desired doses in treatment. Supporting these obtained preclinical data with further studies is very important not only for patients receiving CIS therapy but also in terms of reducing the side effects caused by other chemotherapeutic agents.

## AUTHOR CONTRIBUTIONS

**Betul Cicek:** Conceptualization (equal); methodology (equal); writing – original draft (equal). **Betul Danisman:** Formal analysis (equal); methodology (equal); writing – original draft (equal). **Ismail Bolat:** Formal analysis (equal); methodology (equal); writing – original draft (equal). **Metin Kiliclioglu:** Formal analysis (equal); methodology (equal); writing – original draft (equal). **Mehmet Kuzucu:** Investigation (equal); visualization (equal); writing – original draft (equal). **Halis Suleyman:** Visualization (equal); writing – original draft (equal). **Konstantinos Tsarouhas:** Investigation (equal); visualization (equal); writing – original draft (equal). **Aristidis Tsatsakis:** Supervision (equal); writing – original draft (equal); writing – review and editing (equal). **Ali Taghizadehghalehjoughi:** Conceptualization (equal); investigation (equal); supervision (equal); writing – original draft (equal).

## FUNDING INFORMATION

This research did not receive any specific grant from funding agencies in the public, commercial or not‐for‐profit sectors.

## CONFLICT OF INTEREST STATEMENT

The authors declare no conflicts of interest.

## Data Availability

Data will be made available on request.
